# Clinical Outcome Using Different Catheter Interventional Treatment Modalities in High-Risk Pulmonary Artery Embolism

**DOI:** 10.3390/jcdd11070228

**Published:** 2024-07-22

**Authors:** Luise Antonia Mentzel, Parham Shahidi, Stephan Blazek, Dmitry Sulimov, Holger Thiele, Karl Fengler

**Affiliations:** Department of Cardiology, Heart Center Leipzig at University of Leipzig, Struempellstr. 39, 04289 Leipzig, Germany; luise.mentzel@medizin.uni-leipzig.de (L.A.M.); parham.shahidi@medizin.uni-leipzig.de (P.S.); stephan.blazek@medizin.uni-leipzig.de (S.B.); dmitry.sulimov@medizin.uni-leipzig.de (D.S.); holger.thiele@medizin.uni-leipzig.de (H.T.)

**Keywords:** pulmonary artery embolism, high-risk PE, catheter-directed therapy, catheter embolectomy, thrombectomy, large-bore, intensive care, obstructive shock

## Abstract

Background: For patients with high-risk pulmonary artery embolism (PE), catheter-directed therapies pose a viable alternative treatment option to systemic thrombolysis or anticoagulation. Right now, there are multiple devices available which have been proven to be safe and effective in lower-risk settings. There is, however, little data comparing their efficacies in high-risk PE. Methods: We performed a retrospective, single-center study on patients with high-risk PE undergoing catheter interventional treatment. Patients receiving large-bore catheter thrombectomy were compared to patients receiving alternative treatment forms. Results: Of the 20 patients included, 9 received large-bore thrombectomy, and 11 received alternative interventional treatments. While the baseline characteristics were comparable between the two groups, periprocedural and in-hospital mortality tended to be significantly lower with large-bore thrombectomy when compared to other treatment forms (0 vs. 55% and 33 vs. 82%, *p* = 0.07 and 0.01, respectively). Conclusions: In this small, retrospective study, large-bore thrombectomy was associated with lower mortality as compared to alternative treatment forms. Future prospective research is needed to corroborate these findings.

## 1. Introduction

Patients with acute pulmonary artery embolism (PE) show a broad range of symptoms. While some only present with little to no complaints, in others, PE can result in obstructive shock and a substantially elevated risk of death. In these patients, PE is classified as high-risk according to the current European Society of Cardiology (ESC) guidelines [1].

Guideline-directed therapy suggests systemic thrombolysis as the first-line therapy for patients with PE and obstructive shock. If systemic thrombolysis fails or is contraindicated, catheter interventional therapies are considered an alternative treatment option [1].

While there is, overall, encouraging data for interventional treatment in high-risk PE [2], data comparing the efficacy of different catheter interventional treatment forms in high-risk PE is scarce. Furthermore, these patients are often excluded from clinical trials (i.e., clinicaltrials.gov NCT04790370 and NCT05111613).

When treating high-risk patients in cardiogenic shock, relieving pulmonary artery thrombus as quickly and completely as possible should translate to improved mortality outcomes. As large-bore devices like the FlowTriever^®^ (FT) (Inari Medical, Irvine, CA, USA) present with greater internal diameters (16–24 French) than other devices, this might be the most effective method of thrombus extraction, possibly translating to faster recovery and ultimately affecting mortality.

We aimed to investigate outcomes with large-bore thrombectomy as compared to other methods for high-risk pulmonary embolism in a retrospective, single-center cohort.

## 2. Materials and Methods

Retrospective data were obtained from our hospital’s archives. These contained information on clinical characteristics, lab results, and interventional and echocardiographic data, as well as saved images for each individual case. All the available information was analyzed by the same physician. This retrospective study was conducted in accordance with local ethical regulations and the Declaration of Helsinki. 

### 2.1. Patient Selection

**Figure 1:**
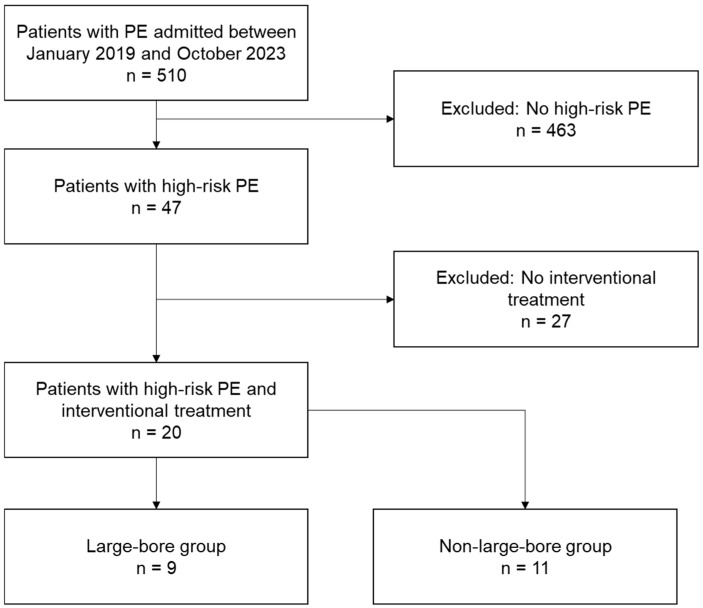
Flowchart depicting patient selection for this study.

All patients who presented with pulmonary artery embolism at the Heart Centre Leipzig at Leipzig University from January 2019 to October 2023 were risk-stratified by using the current ESC guidelines [1] and pulmonary severity index (PESI) score [1]. High-risk PE was defined by hemodynamic instability, i.e., the presence of shock or ongoing hypotension requiring vasopressors when presenting with acute pulmonary artery embolism. Only high-risk PE patients treated with interventional therapy were included in this study (as seen in Figure 1).

### 2.2. Interventional Treatment

As this is a retrospective study, the decision on which device to use was made by the treating interventional cardiologists on an individual basis, depending on the availability of devices. While non-large-bore catheters (local fibrinolysis with EKOS™ [Boston Scientific, Marlborough, MA, USA; 6 French], Indigo™ thrombectomy [Penumbra, Alameda, CA, USA; 8 French], and sheath-based thrombectomy [8–12 French]) were available throughout the entire study period at our center, FlowTriever^®^ (FT) (Inari Medical, Irvine, CA, USA), as a large-bore thrombectomy system, has only been available since 2021. FlowTriever was the only large-bore thrombectomy device used at our center. 

### 2.3. Clinical Endpoints

Clinical parameters (heart rate, systolic and diastolic blood pressure, breathing rate, and body temperature) were assessed before and around 24 h after therapy. Echocardiography, if available, was used to determine the RV/LV ratio and the estimated systolic pulmonary artery pressure before and after therapy. The RV/LV ratio was obtained from the right and left ventricular end-diastolic diameters measured through cardiac ultrasound or, if this was not available, through CT imaging. Intrahospital and periprocedural mortality were assessed separately.

### 2.4. Statistics

The statistical results were acquired using SPSS Version 29.0.0 (IBM, Chicago, IL, USA). For between-group comparisons in binary baseline characteristics, a two-sided Fisher’s exact test was used. For differences in continuous data, two-sided *t*-tests were used for independent samples. Continuous data are presented as mean ± standard deviation. Within-group comparisons of continuous parameters before and after therapy were obtained through a paired-sample *t*-test. Graphs were built via Microsoft Excel Version 16.0 (Microsoft, Redmond, WA, USA).

## 3. Results

After risk stratification and the exclusion of all high-risk PE patients treated by systemic anticoagulation or fibrinolysis only, a total of twenty patients (mean age: 64 ± 16 years) with high-risk PE and interventional treatment were identified (Figure 1). All the patients included met the criteria for cardiogenic shock. The contraindications for systemic fibrinolysis were prolonged cardiopulmonary resuscitation (CPR) (i.e., >60 min) in seven patients; frailty with high clinical bleeding risk in six patients (i.e., liver cirrhosis, known carcinoma with history of bleeding, acute multi-organ failure, or acute severe thrombocytopenia); previous surgery in five patients; failed systemic fibrinolysis in three individuals; acute gastrointestinal or intrabronchial bleeding in three patients; bleeding diathesis upon extracorporeal membrane oxygenation (ECMO) in two patients; and thrombus in transit, conservatively treated acute bone fracture, and unknown reasons, each in one subject. For some of the patients, more than one of the above-mentioned contraindications was present at admission.

Out of these twenty patients, nine (45%) were treated with large-bore thrombectomy via the FlowTriever device and eleven (55%) with other catheter-based methods (nonFT group: EKOS™ [Boston Scientific, Marlborough, MA, USA; 6 French], Indigo™ thrombectomy [Penumbra, Alameda, CA, USA; 8 French], and sheath-based thrombectomy [8–12 French]). In the nonFT cohort, four individuals (36%) received treatment through a combination of EKOS™ and sheaths. In one patient (9%), an EKOS™ catheter alone was used, and another four patients were handled with sheaths alone. The Indigo™ device was used in two patients (18%).

### 3.1. Baseline Parameters

#### 3.1.1. Patient Characteristics

The baseline characteristics were comparable between both cohorts, except for body mass index (BMI), which was higher in the FT group. There was also a trend towards a higher age and more female patients in this cohort (Table 1).

#### 3.1.2. Clinical Features

When comparing clinical traits, numerical differences between groups were observed: Five patients (46%) in the nonFT group and three (33%) in the FT group presented with initial cardiopulmonary resuscitation before intervention. Seven patients (64%) in the nonFT group and four patients (44%) in the FT group needed mechanical ventilation at admission, and six (55%) versus three individuals (33%) underwent veno-arterial extracorporeal membrane oxygenation (ECMO) therapy. 

The pulmonary severity index (PESI) score was comparable between the two groups at around 160 points (*p* = 0.74), which places them at the highest stage of early mortality risk [3]. The baseline lactate values were comparable between the groups (Table 2).

### 3.2. Clinical Endpoints

Before interventional treatment, all patients presented with tachycardia, an elevated breathing rate, and hypotension as signs of cardiogenic or obstructive shock. The RV/LV ratio was elevated as a sign of acute right ventricular pressure overload in both cohorts. 

**Figure 2:**
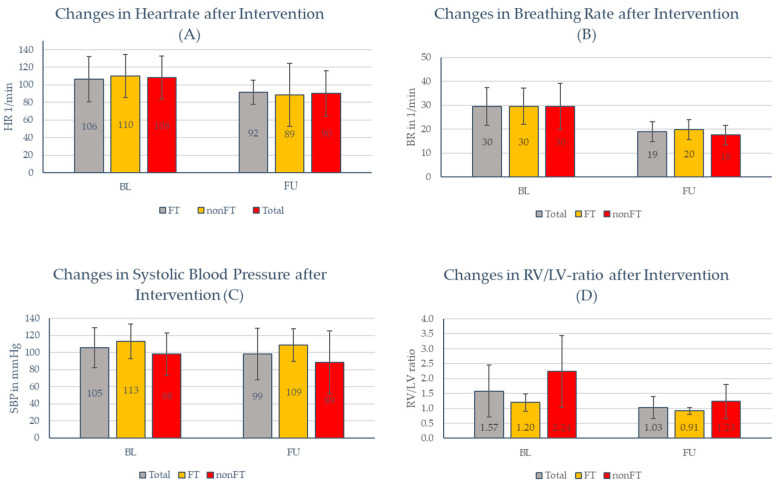
All values are depicted as mean ± standard deviation. (**A**) *p*-values (*t*-test): total: *p* = 0.03; FT: *p* = 0.05; and nonFT: *p* = 0.12; (**B**) *p*-values (*t*-test): total: *p* = 0.001; FT: *p* = 0.01; and nonFT: *p* = 0.06; (**C**) *p*-values (*t*-test): total: *p* = 0.16; FT *p* = 0.34; and nonFT: *p* = 0.17; and (**D**) *p*-values (*t*-test): total: *p* = 0.004; FT *p* = 0.02; and nonFT: *p* = 0.03.

After interventional treatment, the RV/LV ratio, heart rate, and breathing rate were significantly reduced for the entire cohort (*p* < 0.05 for all, Figure 2A–D; Appendix A Table A1, Table A2 and Table A3), while systolic blood pressure was unchanged. When comparing patients in the FT group with those in the nonFT group, no significant differences were observed between the two groups (Figure 2A–D). Notably, due to the high periprocedural mortality in the nonFT group, follow-up values were available for a lesser number of patients.

### 3.3. Mortality Outcome

Six patients (30%) died during or immediately after the intervention. Periprocedural mortality tended to be significantly higher in the nonFT group [six (54.5%) vs. none (0%), *p* = 0.07] compared to the FT group. 

**Figure 3:**
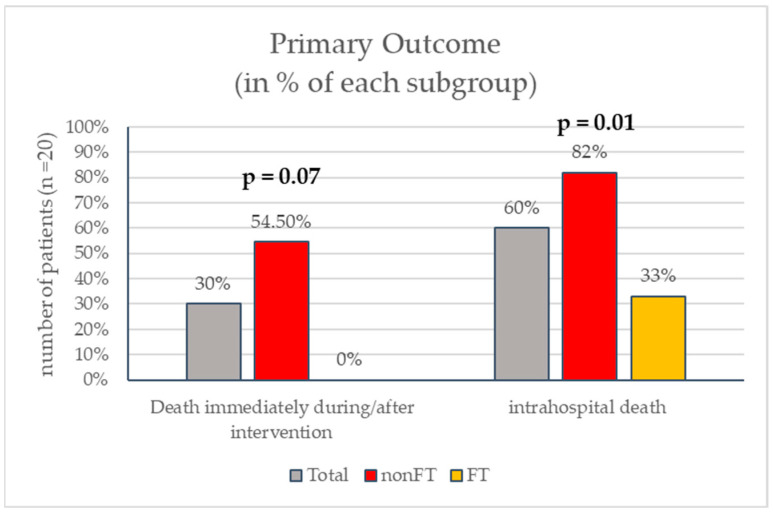
*p*-values for between-group comparison.

Intrahospital death occurred in twelve (60%) patients and was also higher with nonFT devices: nine patients (82%) in the nonFT group versus three (33%) in the FT group (*p* = 0.01) died during hospitalization (Figure 3).

## 4. Discussion

To the best of our knowledge, this is the first comparison of large-bore vs. non-large-bore catheter interventional treatment in patients with high-risk pulmonary embolism. Our preliminary data suggest improved survival with large-bore thrombectomy as compared to other treatment modalities. 

Not only did we observe a trend towards a decline in periprocedural mortality, but we also found significantly lower all-cause intrahospital mortality. The changes in clinical parameters as surrogates for hemodynamic stability and burden of disease (Appendix A, Table A1, Table A2 and Table A3) also widely support our hypothesis that these at-risk patients especially benefit from larger catheter diameters and a fast reperfusion strategy. 

Currently, most prospective research concerning catheter-directed treatment (CDT) options represents lower-risk settings (i.e., intermediate–high-risk patients) [4,5,6,7]. However, the safety and effectiveness of the FlowTriever device in the high-risk subgroup have also been shown in different studies [2,8]. Exemplary results from the FLAME study (FlowTriever for acute massive PE) showed a significant mortality benefit of FlowTriever vs. systemic thrombolysis or anticoagulation (in-hospital mortality 2% vs. 30%) [2] for high-risk patients. However, this was not a randomized trial, and the comparison of outcomes was only made for systemic thrombolysis as an alternative treatment option. This is found throughout most randomized prospective research for CDT (i.e., FLAME, SEATTLE II, CANARY [2,4,9]) since anticoagulation is still the recommended first-line treatment [1]. Additionally to the above-mentioned findings from the FLAME study, our analysis suggests that if the decision to use interventional reperfusion therapy is made, large-bore devices should probably be favored among the multiple treatment options for high-risk PE patients. 

Since the current guidelines do not suggest a distinctive device choice, right now, these choices are left to the treating physician or, at best, to multidisciplinary so-called PERTs (pulmonary embolism response teams) [1,10]. This data review helps build evidence for future decision making for these ultimately at-risk patients.

For now, studies comparing catheter devices to each other in this subgroup are scarce. However, one nationwide US database analysis interestingly reports quite opposing results: their comparison of all-cause hospital mortality in high-risk PE between catheter-directed thrombolysis (CDT) and catheter-directed thrombectomy (CDE) shows no significant difference between both device options (39.6 vs. 34.2%, *p* = 0.07) [11]. These reported mortality rates for both groups are comparable to the outcomes we showed in our FT cohort (Figure 3). However, there were some major differences to our analysis. Firstly, patients were retrospectively included from a registry by using ICD-10 codes and not chosen according to actual clinical parameters. Therefore, only 48.2% of the patients met the clinical criteria for cardiogenic shock out of the all the patients included in our analysis. This suggests a major difference in the burden of disease and hemodynamic stability between the analyzed groups. All of our patients would fall into a category that is described as (super-) massive or ‘catastrophic’ PE [12] in the recent literature, and worse outcomes would be expected in the first place. Secondly, there was no difference made in their CDE group between large-bore and smaller devices. Catheter sizes were not assessed. Interestingly, the mortality rate displayed in this registry for high-risk PE with any catheter-directed treatment was only 36.2% [11], while in Germany, those rates from a recent nationwide analysis are as high as 64.4% [13] for patients treated with CDE, which is more comparable to what we observed. Also, the all-cause in-hospital mortality rates for all the high-risk PE patients showed different results (from 38 to 48% in the US cohort [11,14] vs. 77% in the German population [13]). These numbers strongly suggest that there might be significant differences in classifying these ‘high-risk’ patient groups with the US registries. The total incidence of acute PE was comparable between the studies. These observations additionally lead to the conclusion that our results depict an even smaller percentage of patients with an even higher expectancy of PE-related death (so-called ‘catastrophic PE’). 

When discussing mortality rates, they were exceedingly high in the overall cohort, especially in the nonFT group. This again reflects the ultima ratio situation many patients in this cohort presented with, and we cannot fully exclude the presence of negative selection bias, with different results for non-large-bore devices in less critical patients. Nevertheless, the high-risk PE mortality rates from a recent German nationwide analysis for hemodynamically unstable patients were as high as 77% and about 84% when CPR was needed [13]. This is again comparable to the results we observed for the nonFT group. In the FT cohort, although CPR was also administered in 33% of patients, we saw a much lower in-hospital mortality. This again points towards a potentially better outcome with FT. 

The small group of patients analyzed here was at an extremely high risk of death and in severe obstructive shock, with 45% requiring VA-ECMO treatment. Patients in such conditions might have the most to gain from the fast removal of central thrombotic material, especially since high clot burdens at the central vasculature contribute to pulmonary resistance more severely than a comparable amount of peripheral clots, which are usually out of reach for the FT device anyway and are more amenable to local fibrinolytic treatment [15].

Even though there were numerical differences between the FT and nonFT cohorts concerning the use of VA-ECMO therapy (Table 2), this supposedly did not have a relevant influence on their outcome. A late meta-analysis conducted from 29 observational studies showed no difference in short-term survival when VA-ECMO was used [14], while another series of case reports [15] even showed a possible benefit of VA-ECMO being used when a reperfusion strategy was administered. Although a positive outcome was only shown for surgical embolectomy, the guidelines today cautiously suggest the use of VA-ECMO in carefully selected patients [1], with the possible benefit of VA-ECMO being discussed in other analyses, including one from 2022 based on a national German database [16,17,18]. Therefore, the numerical differences in mechanical support therapy between the compared groups might be less relevant.

### 4.1. Limitations

There are certain limitations to our study which need to be mentioned. First, the data were only obtained retrospectively. The choice of device was individually made by the treating physician, and patients were not randomized between groups. Therefore, the treated groups were not homogeneous, and any statistics should be regarded with caution. Follow-up was lost after discharge from the clinic due to the lack of a prospective design. Also, selection bias has to be considered as a potential confounder. 

Secondly, the number of patients in this analysis was very low. When checking the German nationwide analysis mentioned above, only 8.9% of all patients with PE were hemodynamically unstable, and only 3.5% were in shock [13]. Another prior cohort analysis published in 2018 also reports a percentage of 3.5% for hemodynamic instability [19]. Out of just over 500 patients with acute PE, who presented at our clinic during the time frame of our analysis, only a fraction were high-risk patients (as seen in Figure 1). Out of those, all were treated according to current guidelines at the time of admission. This allowed catheter-based thrombectomy to be administered to only a handful of high-risk patients as a bail-out strategy when systemic thrombolysis was contraindicated or had already failed. Even though our clinic is a high-volume tertiary care center for patients with PE and many are referred from smaller hospitals, the number of patients fitting the inclusion criteria of this analysis was still low (Figure 1). To increase numbers and obtain more impactful statistical data, a multicenter approach is definitely necessary.

Thirdly, the clinical baseline characteristics were not perfectly balanced between the groups; in particular, BMI was different between the two groups. Even though a higher BMI is usually associated with a worse outcome, we cannot exclude that this affected the outcomes.

Our results, as presented above, can, due to the low number of patients, only be seen as hypothesis-generating. Future research, ideally prospective randomized trials, is needed to validate this hypothesis.

### 4.2. Conclusions

In conclusion, improved outcomes with large-bore thrombectomy when compared to other device-based approaches in high-risk PE patients, especially those in obstructive shock, may be plausible. This is corroborated by our data on all-cause in-hospital mortality outcomes as well as changes in clinical parameters and burden of disease shown throughout this study. Future research, ideally prospective randomized trials, is needed to validate this hypothesis.

## Figures and Tables

**Table 1:** Baseline Characteristics.

	Total (n = 20)	NonFT (n = 11)	FT (n = 9)	*p*-Value
Age (years)	64 ± 16	61 ± 16	68 ± 16	0.17
Height (cm)	168 ± 14	173 ± 15	163 ± 13	0.13
Weight (kg)	84 ± 23	77 ± 19	91 ± 27	0.09
BMI (kg/m^2^)	30 ± 8	26 ± 6	34 ± 7	0.02
Sex				
Male (n, %)	8 (40)	5 (46)	3 (33)	0.67
Female (n, %)	12 (60)	6 (55)	6 (67)	
CAD (n, %)	3 (15)	2 (18)	1 (11)	0.99
congestive heart disease (n)	0	0	0	0.99
COPD (n, %)	1 (5)	0	1 (11)	0.99
active COVID infection (n, %)	1 (5)	1 (9)	0	0.99
Type II Diabetes (n, %)	2 (10)	1 (9)	1 (11)	0.99
DVT (n, %)	8 (40)	3 (27)	5 (56)	0.36
(suspected) cancer (n, %)	3 (20)	2 (18)	1 (11)	0.99
Smoking (n, %)	5 (25)	3 (27)	2 (22)	0.99
Surgery during last month (n, %)	7 (35)	3 (27)	4 (44)	0.64

patient characteristics at baseline (BMI: body mass index; CAD: coronary artery disease; COPD: chronic obstructive pulmonary disease; DVT: deep vein thrombosis).

**Table 2:** Clinical Characteristics.

	Total (n = 20)	NonFT (n = 11)	FT (n = 9)	*p*-Value
CPR (n, %)	8 (40)	5 (46)	3 (33)	0.67
vasopressors (n, %)	14 (70)	9 (82)	5 (56)	0.34
mechanical ventilation (n, %)	11 (55)	7 (64)	4 (44)	0.65
VA-ECMO (n, %)	9 (45)	6 (55)	3 (33)	0.41
Lactate (mmol/L)	6.5 ± 4.2	6.2 ± 4.8	6.8 ± 3.3	0.78
PESI score	162 ± 40	165 ± 48	158 ± 28	0.74

clinical traits/needed intensive care treatment at admission (CPR: cardiopulmonary resuscitation; VA-ECMO: veno-arterial extracorporeal membrane oxygenation; PESI: pulmonary severity index).

## Data Availability

The raw data supporting the conclusions of this article will be made available by the authors on request.

## Appendix A

**Table A1:** Total cohort clinical Presentation.

	Baseline	Followup	Absolute ∆	*p*-Value
Heart rate (1/min)	108 ± 23	90 ± 26	−18 ± 36	0.03
Respiratory rate (1/min)	30 ± 8	19 ± 4	−11 ± 8	0.001
SBP (mmHg)	105 ± 23	99 ± 30	−6 ± 27	0.16
DBP (mmHg)	59 ± 9	58 ± 15	−1 ± 15	0.40
RV/LV ratio	1.5 ± 0.7	1.1 ± 0.4	−0.4 ± 0.5	0.008

clinical presentation of all patients.

**Table A2:** nonFT cohort clinical presentation.

	Baseline	Followup	Absolute ∆	*p*-Value
Heart rate (1/min)	110 ± 24	89 ± 36	−21 ± 47	0.12
Respiratory rate (1/min)	30 ± 10	18 ± 4	−12 ± 11	0.06
SBP (mmHg)	98 ± 25	89 ± 37	−9 ± 27	0.17
DBP (mmHg)	56 ± 12	55 ± 17	−1 ± 19	0.42
RV/LV ratio	1.8 ± 0.85	1.2 ± 0.5	−0.6 ± 0.6	0.05

clinical presentation of nonFT cohort.

**Table A3:** FT cohort clinical presentation.

	Baseline	Followup	Absolute ∆	*p*-Value
Heart rate (1/min)	106 ± 26	92 ± 14	−14 ± 22	0.05
Respiratory rate (1/min)	30 ± 8	20 ± 4	−10 ± 8	0.01
SBP (mmHg)	113 ± 20	109 ± 19	−4 ± 29	0.34
DBP (mmHg)	61 ± 5	61 ± 13	0 ± 11	0.45
RV/LV ratio	1.2 ± 0.3	1.0 ± 0.2	−0.2 ± 0.3	0.03

clinical presentation of FT cohort.
